# Condemned Food

**Published:** 1875-02

**Authors:** 


Condemned Food.—That much food is swallowed which never should be taken into the stomach, is beyond doubt. There is hardly a table any where on which some dish is not placed, the contents of which were far better thrown away than eaten. This is generally the most delicate and appetizing dish of the day; the most highly-seasoned, and the richest. But while it would be a measure of sanitary safety for a competent inspector to visit the tables of the majority about dinner-time and dispose of useless articles, we hardly expect to see the system introduced. The lives of men and women will still be worn out in the preparation of useless and even injurious food, and dyspepsia and numerous irregularities of the interior department will still be common. Indeed it is doubtful whether we shall ever have such a complete system of inspection of the raw materials offered for sale, as will save us from the danger of eating unsound articles. In London, the police supervision, in this direction, is far more rigid. In the report of the inspectors of food for the City of London, recently presented to the corporation, it is stated that, in the year preceeding, there had been condemned nearly 80 tons of meat, more than 1,000,000 fish, weighing 400 tons, 4,600 pounds of eels, about 2,000 bushels of shrimps, sprats, oysters, periwinkles, whelks, mussels, and cockles. Fruit appear, to be as objectionable as fish, for, to say nothing of cocoa-nuts and other delicacies seized in the streets, there were condemned in bonded warehouses, 30 hogsheads, 896 boxes, 600 barrels, 40 bags, and 69 cart-loads of figs ; and, in the same warehouses, boxes and barrels of currants underwent the same fate. It is matter for satisfactory reflection, in respect to the public health, that legal inspection prevented this vast mass of disease-breeding filth from being offered for sale, and that the figs not sold in the streets were also prevented from being converted, by dishonest experts, into jam or some other "delicacy for invalids."

---

Digestion begins in the mouth. If the mouth fails to do its part, the stomach is over-burdened. Hard food is better than soft, because it stays longer in the mouth, and is thus better prepared.

